# Structural Dynamics of the SARS-CoV-2 Spike Protein: A 2-Year Retrospective Analysis of SARS-CoV-2 Variants (from Alpha to Omicron) Reveals an Early Divergence between Conserved and Variable Epitopes

**DOI:** 10.3390/molecules27123851

**Published:** 2022-06-15

**Authors:** Patrick Guérin, Nouara Yahi, Fodil Azzaz, Henri Chahinian, Jean-Marc Sabatier, Jacques Fantini

**Affiliations:** 1 OpenHealth—CP 130—CEDEX, 56038 Vannes, France; p.guerin@openhealth.fr; 2 INSERM UMR_S 1072, Aix-Marseille University, CEDEX, 13015 Marseille, France; nouara.y@gmail.com (N.Y.); azzaz.fodil@gmail.com (F.A.); henrichahinian@gmail.com (H.C.); 3 Inst Neurophysiopathol, Aix-Marseille University, CNRS, INP, CEDEX, 13005 Marseille, France; sabatier.jm1@gmail.com

**Keywords:** SARS-CoV-2 variants, vaccine, facilitating antibodies, neutralizing antibodies, molecular epidemiology

## Abstract

We analyzed the epitope evolution of the spike protein in 1,860,489 SARS-CoV-2 genomes. The structural dynamics of these epitopes was determined by molecular modeling approaches. The D614G mutation, selected in the first months of the pandemic, is still present in currently circulating SARS-CoV-2 strains. This mutation facilitates the conformational change leading to the demasking of the ACE2 binding domain. D614G also abrogated the binding of facilitating antibodies to a linear epitope common to SARS-CoV-1 and SARS-CoV-2. The main neutralizing epitope of the N-terminal domain (NTD) of the spike protein showed extensive structural variability in SARS-CoV-2 variants, especially Delta and Omicron. This epitope is located on the flat surface of the NTD, a large electropositive area which binds to electronegatively charged lipid rafts of host cells. A facilitating epitope located on the lower part of the NTD appeared to be highly conserved among most SARS-CoV-2 variants, which may represent a risk of antibody-dependent enhancement (ADE). Overall, this retrospective analysis revealed an early divergence between conserved (facilitating) and variable (neutralizing) epitopes of the spike protein. These data aid in the designing of new antiviral strategies that could help to control COVID-19 infection by mimicking neutralizing antibodies or by blocking facilitating antibodies.

## 1. Introduction

More than two years after the beginning of the COVID-19 pandemics, the evolution of SARS-CoV-2 is still puzzling, one variant after another replacing its predecessor, with no clearcut logic. This constantly moving situation is not optimal for securing therapeutic and/or preventive strategies [1]. Most medical efforts have been devoted to mass vaccination, a first encounter with an RNA virus with a high mutation potential. In the fight against viruses, the immune system has two main weapons, cytotoxic T-cells and neutralizing antibodies, both playing a key role in the control of viral infections, especially in the case of respiratory viruses [2,3]. However, virus-specific antibodies can also promote pathology, a phenomenon referred to as antibody-dependent enhancement (ADE) [4]. ADE of virus infection is generally due to virus-specific antibodies that facilitate the entry of the virus into host cells, and in some cases, increase virus replication in monocytes, dendritic cells and macrophages through antibody binding to Fcγ receptors [5]. In addition, alternative mechanisms of ADE involving the complement component C1q have been reported [6]. ADE has been observed in two typical situations: (i) reinfection with a virus variant after primary infection with a different strain [7] or a cross-reactive virus [8], and (ii) as the result of viral infection in vaccinated people [9]. The ADE phenomenon was initially discovered in flaviviruses in the late 1960s [10] and experimentally demonstrated in the early 1970s [11]. It concerns a broad range of viruses including dengue [12], Ebola [13], Zika [14], HIV [15], influenza [16], and various animal and human coronaviruses [17].

As early as in June 2020, at a time when COVID-19 vaccines had just entered clinical evaluation, Akiko Iwasaki and Yexin Yang from Yale University School of Medicine alerted that “ADE should be given full consideration in the safety evaluation of emerging candidate vaccines for SARS-CoV-2” [18]. A similar warning on vaccine safety due to potential risks of ADE was independently published by Shibo Jiang [19]. In contrast, several authors considered the risk to be null or minimal in the case of SARS-CoV-2 [20,21,22,23].

However, several pieces of evidence may argue in favor of an ADE issue for SARS-CoV-2: (i) ADE has been reported for animal coronaviruses such as feline infectious peritonitis virus [24]. In the most dramatic cases, kittens previously vaccinated with a recombinant virus containing the spike protein gene succumbed to early death after a coronavirus challenge [25]; (ii) ADE epitopes were characterized in the spike protein of this feline coronavirus [26] (iii) ADE epitopes have also been found in human coronaviruses related to SARS-CoV-2, i.e., SARS-CoV-1 [27] and MERS-CoV [28,29]. The case of SARS-CoV-1 is particularly interesting since its spike protein displays a linear ADE epitope, 597-LYQDVNC-603 (recognized by the monoclonal antibody 43-3-14) [27] that is fully conserved in the SARS-CoV-2 spike protein sequence used for mRNA COVID-19 vaccines; (iv) ADE antibodies directed against the N-terminal domain (NTD) of the spike protein have been detected and characterized in convalescent COVID-19 patients [30,31]. Indeed, Okuya et al. showed that ADE antibodies were found in 41.4% of the acute COVID-19 patients [32]; and (v) ADE antibodies are suspected to be particularly efficient in vaccinated COVID-19 patients infected with the Delta variant [33,34]. In this context, we recently reported that facilitating anti-spike antibodies targeting the NTD have a higher affinity for the Delta variant than for the initial Wuhan strain [33]. We also reported that the main neutralizing epitope of the NTD is almost lost in Delta variants [33]. This finding is of critical importance since ADE infection of coronaviruses is known to be induced by the presence of sub-neutralizing levels of anti-spike antibodies [35]. Overall, these data suggest that the balance between neutralizing and facilitating antibodies may differ greatly according to the virus strain [33].

The main objective of the present study was to try to understand the evolution of neutralizing and facilitating epitopes since the beginning of the COVID-19 pandemic. To this end, we analyzed a panel of representative SARS-CoV-2 variants [36] including Alpha, beta, gamma, Delta, lambda, mu, and Omicron. We used multiple amino acid sequence alignment methods combined with molecular modeling approaches to determine the structural dynamics of selected epitopes in the SARS-CoV-2 spike protein.

## 2. Methods

The mutational pattern of SARS-CoV-2 variants was extracted as an Excel file from the Los Alamos database (https://cov.lanl.gov/components/sequence/COV/int_sites_tbls.comp, accessed on 29 November 2021). Molecular modeling studies were performed with the Hyperchem [37] and Deep View/Swiss-Pdb viewer [38] programs, as described in previous studies [36,39,40,41,42,43]. Special attention was given to the regions that are unresolved in pdb files [44], especially the 621-640 loop. To overcome this problem, a complete structure of the original SARS-CoV-2 spike protein encompassing residues 14-1200 was generated and characterized as described previously [45]. Minimized structures of the spike protein of each variant were then obtained by introducing appropriate mutations and/or deletions in the complete spike protein. Energy minimizations of the variants were performed with the Polak-Ribière conjugate gradient algorithm with the Bio-CHARMM force field in Hyperchem using a maximum of 3 × 10^5^ steps and a root mean square (RMS) gradient of 0.01 kcal/mol.Å as the convergence condition [46]. The energy of interaction (∆G) of each antibody-spike protein complex was calculated with the Molegro Molecular Viewer [47]. The cluster of gangliosides GM1 in a typical lipid raft organization was generated as described previously from the CHARMM-GUI Glycolipid Modeler [48] and submitted to several minimization steps with the Polak-Ribière algorithm [46].

## 3. Results

### 3.1. Mutational Landscape of SARS-CoV-2 Spike Protein

The mutational patterns and geographic origins of the SARS-CoV-2 variants analyzed in this study are summarized in Table 1. All variants have a dual nomenclature (lineage and Greek letter) except for C.1.2, a variant that emerged in South Africa in the summer of 2021 [49]. Our analysis is focused on the NTD and on the rod-like domains of the spike protein. Other ADE and neutralization epitopes do exist in the RBD, but during the complex process of viral adhesion to target cells, this domain is involved at a later step [36,41]. Clearly, the NTD is key to understanding how SARS-CoV-2 initially interacts with the plasma membrane of host cells.

### 3.2. Structural Analysis of Mutation D614G and Its Relationship with a Linear ADE Epitope

**Figure 1:**
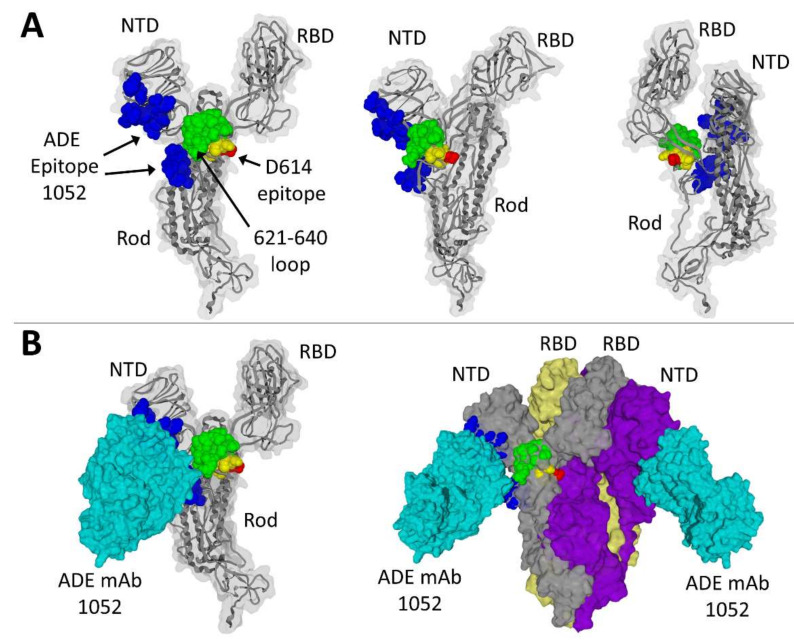
Localization of ADE epitopes on the spike protein. (**A**) Three distinct views of the SARS-CoV-2 spike protein (Wuhan strain). The discontinuous ADE epitope recognized by the 1052 antibody (ADE mAb) is colored in blue. The common coronavirus ADE epitope (D614 epitope) is colored in yellow, with amino acid residue D614 in red. The 621-640 loop that is missing in PDB: 7LAB is in green. (**B**) ADE antibody 1052 (in cyan) bound to the monomeric spike (left panel) or to the trimeric spike protein (right panel). The N-terminal domain (NTD) and receptor-binding domain (RBD) are indicated in all models.

D614 belongs to the 611-617 amino acid sequence LYQDVNC, a linear ADE epitope recognized by the 43-3-14 antibody [27]. This epitope is common to human coronaviruses SARS-CoV-1 and SARS-CoV-2. Interestingly, it is centered on position 614, which is an aspartic acid residue in the original Wuhan strain but has rapidly evolved to the ultra-dominant D614G during the first months of 2020 [50]. The localization of this epitope on the spike protein (Wuhan strain) is shown in Figure 1A (epitope colored in yellow, except for D614 highlighted in red). It is well exposed on the protein surface so that it can be recognized by facilitating antibodies generated during previous coronavirus infections in humans, especially in geographic areas previously exposed to SARS-CoV-1.

### 3.3. Structural Analysis of a Three-Dimensional ADE Epitope

A second ADE epitope targeted by facilitating antibodies is divided in two parts (both colored in blue in Figure 1A): one in the NTD (27-32, 64-69 and 211-218 segments) and the other one in the rod-like domain (600-607, 674-677 and 689-691 segments) of the spike protein. Antibodies directed against this epitope have been detected in sera from convalescent COVID-19 patients [31]. Although the two parts of this ADE epitope seem to be spatially distant, both are close to a flexible 20-amino acid residue loop (621-640) that is unresolved in PDB files but was added by molecular modeling in the structures shown in Figure 1. It is interesting to note that this loop (highlighted in green) is ideally located to connect the NTD and the RBD, but also to provide a conformational link between both ADE epitopes (Figure 1B).

**Figure 2:**
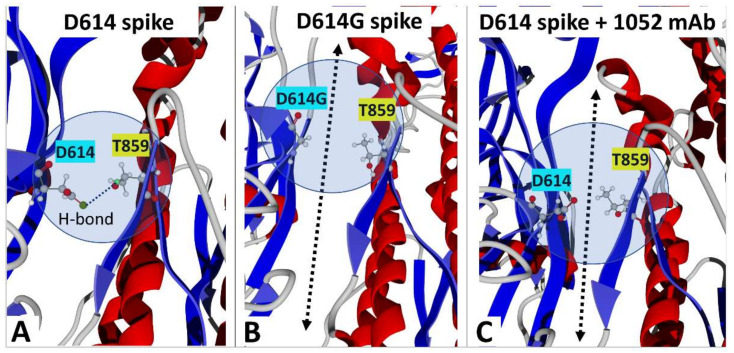
How the D614G mutation and the ADE antibody 1052 enhance SARS-CoV-2 infectivity. (**A**) Hydrogen bond between D614 (chain B) and T859 (chain C) stabilizing the trimeric spike protein (PDB: 6VSB). (**B**) The D614G mutation renders impossible the formation of the hydrogen bond and facilitates the conformational change inducing the demasking of the RBD (PDB: 7BNM). (**C**) Binding of ADE antibody 1052 breaks the hydrogen bond between D614 and T859 (PDB: 7LAB). The arrow in panels B and C illustrates the lack of contact between vicinal spike protein monomers in the context of the trimeric association.

Once the NTD is bound to the cell membrane of the host cell, a conformational change unmasks the RBD, which becomes available for a functional interaction with a viral receptor, chiefly ACE2 [36]. This spatial reorganization leads to the open, fusion-compatible conformation of the trimeric spike protein [51]. In the Wuhan strain, the closed conformation of the trimer [52] is stabilized by a hydrogen bond between D614 of one subunit and T859 of its neighbor (respectively chains B and C in Figure 2A). The global spreading of the pandemic during the first months of 2020 has been associated with the breakthrough of the first SARS-CoV-2 variant with a unique mutation in the spike protein, D614G. As shown in Figure 2B, this mutation induces the loss of the hydrogen bond that stabilized the closed conformation. Thus, we analyzed the status of this hydrogen bond in the complex between the facilitating 1052 antibody and the spike protein trimer. As shown in Figure 2C, the antibody has a long-range conformational effect on both D614 and T859, which renders impossible the formation of this hydrogen bond. It is likely that the 621-640 loop, which conformationally connects the 1052 and the 611-617 epitopes, mediates this distal effect. In this respect, it is interesting to note that this facilitation can be induced by two distinct mechanisms: (i) the replacement of aspartic acid by glycine at position 614 (D614G mutation), or (ii) the binding of the ADE antibody 1052 to the original Wuhan spike protein displaying an aspartic acid (D614) at this position.

### 3.4. Analysis of Amino Acid Sequence Variations in ADE and Neutralizing Epitopes during the Global Spreading of the COVID-19 Pandemic

**Figure 3:**
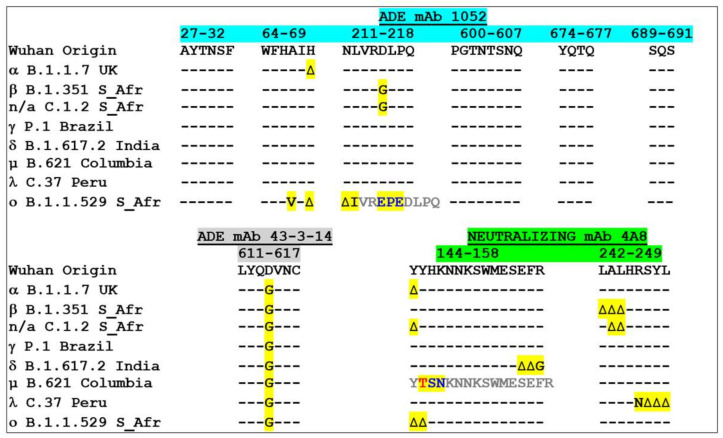
Amino acid sequence alignments of ADE and neutralizing epitopes in SARS-CoV-2 variants. Amino acid residue variations are highlighted in yellow. -, identity; ∆, deletion. Note that the 144-158 neutralizing epitope of the mu (µ) variant displays a threonine residue (T, in red) inserted after Y144, then two mutations after this insertion (colored in blue). The insertion induces a shift of the amino acid sequence (highlighted in grey).

We then analyzed the evolution of the amino acid sequence of ADE epitopes among SARS-CoV-2 variants (Figure 3). The 611-617 epitope (lower left panel), which is common to SARS-CoV-1 and SARS-CoV-2, has a unique signature in all variants, i.e., the D614G mutation. As position 614 is central to the epitope, this epitope is probably no longer recognized by ADE antibodies generated by previous coronavirus infections in humans. The second ADE epitope is formed by several distinct areas in the NTD and in the rod-like regions of the spike protein (Figure 3, upper panel). In the NTD, the epitope is divided in three linear segments that represent ca. 80% of the total energy of interaction of the 1052 antibody-NTD complex (as calculated from PDB: 7LAB): 27-32, 64-69 and 211-218 (accounting for 12, 19 and 51% of the energy of interaction, respectively). The complex is further stabilized by auxiliary contacts with the rod-like region of the spike protein (chiefly 600-607, 674-677 and 689-691). Overall, the whole epitope appeared to be extremely well conserved, except at two amino acid residue positions: H69 and D215. Indeed, a deletion (∆H69) is found in the Alpha variant, and D215 is mutated in D215G in the beta and the more recent C1.2 variants (Figure 3, upper panel). Surprisingly, the recently emerging Omicron variant (BA.1) does not seem to follow this general rule, as its ADE epitope is heavily affected by a combination of single point mutations (A67V, L212I), two deletions (∆H69, ∆N211), and a 3 amino-acid insertion (between R214 and D-215) [43].

In marked contrast with the conservation of the 1052 ADE epitope in most variants, the main neutralizing epitope of the NTD showed extensive amino acid sequence variations (Figure 3, lower right panel). The changes included deletions, insertions and single point mutations that are distributed among two key regions, 144-158 (N3 loop) and 242-249 (N5 loop) that constitute the three-dimensional site recognized by the neutralizing 4A8 antibody [53]. The localization of the neutralization epitope of the NTD at the virus/host cell interface is consistent with this high variability as it is submitted to a strong pressure of selection for SARS-CoV-2 variants. Conversely, the ADE epitope 1052, which is on the lower part of the NTD, is not facing the plasma membrane of the host cell and for this reason it is not subjected to such a high selective pressure.

The frequency of amino acid sequence variations of the ADE and neutralizing epitopes was analyzed by specific queries of the Los Alamos database over a six-month period starting in June 2021 (1 June 2021 to 27 November 2021) (Table 2). All the epitopes listed in Figure 3 were analyzed in 1,860,489 genomes. The ADE epitope of the NTD is highly conserved (>98% for all segments), except for the 64-69 motif at position H69 (variation of 5.46% with 1 mutation), mostly reflecting the Alpha variant [54]. The ADE epitope 611-617 displays 1 mutation in 98.70% of cases, consistent with the worldwide dominance of the D614G mutant [55]. The situation of the neutralization epitope of the NTD is by far more complex, in particular for the 144-158 segment, which shows high amino acid sequence variability (frequency of the Wuhan sequence < 0.05%). Remarkably, 92.04% of the sequences have 2 mutations and some viruses with 3, 4 and even 5 mutations currently detected. The second linear segment (242-249) is more conserved (99.22% of sequences are identical to the Wuhan strain), but viruses with up to 4 mutations have been characterized. Interestingly, the amino acid variations of the neutralization epitope are concentrated on positions that are associated with the variants analyzed in the present study: Y144, E156, F157 and R158, in the N3 loop, R246 in the N5 loop (Figure 3).

### 3.5. Estimating the Risks of the Facilitation Phenomenon Depending on the Variant Concerned: A Molecular Modeling Approach

**Figure 4:**
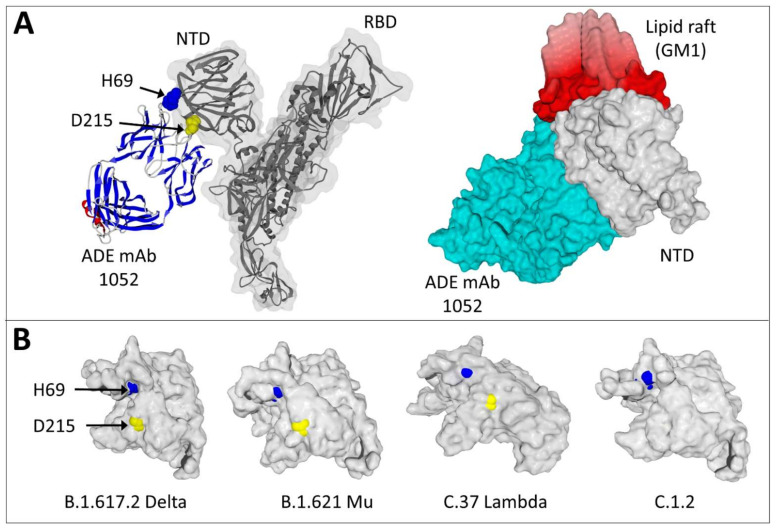
Critical amino acid residues control the binding of the ADE antibody 1052 to variant spike proteins. (**A**) Binding of the 1052 antibody (ADE mAb) to the Wuhan spike protein (PDB: 7LAB) with the NTD and RBD indicated. In the left panel, the light and heavy chains of the antibody are represented in standard secondary structures (red, α-helix, blue, β-strand). H69 (in blue atomic spheres) and D215 (in yellow atomic spheres) are highlighted. In the right panel, a surface representation illustrates the geometric complementarity of the Delta spike protein-antibody complex bound to a cluster of gangliosides GM1 figuring a lipid raft on the plasma membrane of a host cell. Note that the 1052 antibody b simultaneously binds to the NTD of the spike protein and to the edge of the lipid raft. (**B**) Molecular modeling of the NTD of several SARS-CoV-2 variants showing different levels of surface exposure of H69 (in blue) and D215 (in yellow) amino acid residues.

Finally, we used molecular modeling approaches to determine how mutations in ADE epitopes could impair the binding of the facilitating antibodies. In our analysis of ADE epitopes in SARS-CoV-2 variants (Figure 3), we detected two essential mutations that can potentially suppress the facilitation phenomenon: ∆H69 and D215G. Thus, we studied the localization of H69 and D215 in the molecular complex between the ADE antibody 1052 and the spike protein (Figure 4A, left panel). Both H69 and D615 appeared critical for the 1052 antibody binding site on the NTD of the spike protein. These positions are fully conserved in the gamma, Delta, mu and lambda SARS-CoV-2 variants, which are still recognized by the ADE antibody 1052. An illustration of the efficiency of this antibody to facilitate the infection by the Delta variant is shown in Figure 4A (right panel). The plasma membrane of the host cell is represented by a cluster of gangliosides GM1 to figure the lipid raft that acts as a landing platform for the NTD [41]. In line with previous data from our group [33], once the 1052 antibody is bound to the NTD of the Delta spike protein, a global conformation change involving both the NTD and the antibody allows the formation of a highly energetic trimolecular complex (antibody-NTD-lipid raft) with an obvious geometric complementarity of all partners. We then compared the structure of the Delta variant NTD with the mu, lambda, and C.1.2 variants (Figure 4B). Except for C.1.2, which displays a D215G mutation [49], and the highly divergent Omicron [43], (Figure 3) all other variants have both H69 and D215 accessible on the NTD surface.

In line with these data, the energy of interaction of the C.1.2 variant spike protein with the 1052 antibody (−107 kJ·mol^−1^) was less than half the value calculated for the Wuhan strain (−229 kJ·mol^−1^), whereas it reached −246 kJ·mol^−1^ for the Delta variant [33], −236 kJ·mol^−1^ for mu and −228 kJ·mol^−1^ for lambda. Thus, the conservation of H69 and D215 (in gamma, Delta, mu and lambda variants) is critical for virus infectivity, as it may allow an optimal binding of the facilitating 1052 antibody to the spike protein. In contrast, the ADE epitope is affected as soon as at least one of these positions is mutated (as it is the case for the Alpha, beta, C.1.2 and Omicron BA.1 variants). Interestingly, the replacement of H66, W64 and R214 spike amino acid residues by alanine affected the binding of another ADE antibody that targets the lower part of the NTD, which underscores the importance of this region for eliciting facilitating antibodies [30,56].

## 4. Discussion

SARS-CoV-2 evolution has been extensively studied by computational approaches [44,57,58,59,60,61,62]. Our retrospective analysis of SARS-CoV-2 variants revealed an early divergence between conserved (ADE) and variable (neutralizing) epitopes which is based on their localization on the spike protein. If we consider the NTD of the spike protein, the neutralizing antibody binds to the tip of the NTD, on the same flat surface that is recognized by lipid rafts (Figure 4A). In contrast, the facilitating ADE epitope of the NTD is located on the opposite side that is not facing the plasma membrane of the host cell (Figure 4A). Because of these two distinct localizations, the facilitating and the neutralizing epitopes are not submitted to the same evolutionary forces. These data have important implications for therapeutic and vaccine strategies. From a therapeutic point of view, drugs targeting lipid rafts [39] or neutralization epitopes may inhibit SARS-CoV-2 infection [40] if they still bind to currently circulating variants. On the other hand, it might be possible to counteract the facilitating effect of ADE antibodies in both vaccinated and previously contaminated individuals by designing drugs targeting the conserved ADE epitope of the NTD.

A potential drawback of vaccination against viruses is the risk of antibody facilitation (ADE), especially when the strain used for the immunization protocol is distinct from circulating viruses [63]. In the past, ADE has been evidenced for a broad range of human RNA viruses including HIV, influenza, filoviruses, and coronaviruses [5]. Although ADE antibodies have been characterized in the serum from COVID-19 convalescent patients [30,31], the risk of ADE linked to vaccination with spike protein-based vectors (either mRNA or adenovirus) has been minimized, some arguing that ADE antibodies could induce SARS-CoV-2 infection enhancement in vitro but not in vivo [31]. However, a potential caveat of these studies is that SARS-CoV-2 variants have not been specifically assessed. Moreover, surprising higher infection rates in vaccinated vs. unvaccinated individuals in the 0–14 days after the first dose were recently reported in long-term care facility residents and health-care workers, which resulted in significant negative vaccine efficiency estimates of −37% and −113%, respectively [64]. Similarly, Houhamdi et al. reported that 26.3% of patients developed SARS-CoV-2 infection within 21 days following the last dose of vaccine, suggesting possible early production of anti-SARS-CoV-2 facilitating antibodies [65]. To what extent this apparent enhancement of SARS-CoV-2 infection is due (or not due) to an imbalance between vaccine-induced (and/or pre-existing) neutralizing and facilitating antibodies warrants further investigation. Moreover, a recent report revealed that there is no clear relationship between the percentage of fully vaccinated individuals and new COVID-19 cases in 68 countries including Israel, a pioneer in mass vaccination against SARS-CoV-2 [66]. Taken together, these observations suggested that ADE, or more specifically the ADE/neutralization balance, could pose a problem for COVID-19 vaccine strategies, especially during the outbreak of SARS-CoV-2 variants. This potential issue has now been experimentally studied and/or commented on by several authors from different countries [67,68,69,70,71,72,73,74]. Finally, it is worth noting that ADE has been suspected to increase the severity of COVID-19 symptoms in selected geographic areas [75]. In this context, proposing a third and potentially a fourth jab to improve vaccine efficiency to face the threat of a new variant may not be a good idea, as it may further increase the amount of ADE antibodies without a significant gain in neutralizing activity. Instead, we believe that it is now critical to design new vaccine formulations lacking ADE epitopes. Molecular epidemiology surveillance of SARS-CoV-2 coupled with structural analysis of variant spike proteins will certainly help to reach this goal.

In the present study, we focused our attention on two distinct ADE epitopes: one linear epitope common to SARS-CoV-1 and SARS-CoV-2 (611-617 in the rod-like region of the spike protein, recognized by the 43-3-14 antibody) [27] and a complex three-dimensional NTD epitope (recognized by the 1052 antibody) [31]. Both epitopes are present on the spike protein generated by mRNA vaccines as the original formulas are based on the Wuhan strain [76]. Therefore, it is of utmost importance to determine whether these epitopes are still expressed and accessible on SARS-CoV-2 variants. The 611-617 epitope has probably escaped facilitating antibodies because the D614G variant has rapidly replaced the original strain [55]. In the initial study of the D614G mutation, the authors mentioned the presence of D614 in a conserved ADE epitope [50]. Our modeling approaches revealed a common molecular mechanism leading to enhanced infectivity for the D614G variant and for ADE antibodies with the Wuhan strain (Figure 2). In both cases, the loss of a stabilizing hydrogen bond between amino acid residues 614 and 859 of two vicinal spike protein chains relaxes the trimer and facilitates the conformational change that unmasks the RBD. A similar conformational mechanism has been described for another facilitating antibody that also binds to the lower part of the NTD [30,56]. In this respect, a major outcome of our study is the identification of the 621-640 loop, which is missing in PDB files, as a conformational transmitter that allows the 1052 antibody to induce distant effects on amino acid residue 614. The enhancement of infection provided by this ADE antibody involves two distinct Fcγ-independent mechanisms: a long range conformational effect and a stabilization of the NTD bound to a lipid raft [33]. These mechanisms are not mutually exclusive since the 1052 antibody binds simultaneously to the NTD and to the edge of the lipid raft (Figure 4B).

From an epidemiologic point of view, we can propose a scenario according to which the first cases of SARS-CoV-2 infections in China (prior to vaccination) could have been facilitated by ADE antibodies directed against the 611-617 epitope in individuals previously infected by SARS-CoV-1 or similar coronaviruses. This notion is supported by the recent demonstration that non-neutralizing antibodies directed against SARS-CoV-1 and recognizing that the SARS-CoV-2 spike protein may persist for at least 15 years [77]. By then the global extension of SARS-CoV-2 has probably levied this constraint by selecting the D614G variant in SARS-CoV-1 free populations. This scenario is consistent with the rapid raise and long-term maintenance of D614G worldwide. It is also consistent with the discrepancy between the severity of COVID-19 cases observed in the Hubei province of China and those occurring elsewhere in the world at the beginning of the pandemic [75]. Moreover, it explains why ADE has not been detected during the first months following mass vaccination, since ADE antibodies directed against the 611-617 epitope are no longer active on D614G variants. The observation that anti-SARS-CoV-1 antibodies isolated from a convalescent patient could enhance virus infection mediated by civet virus spike proteins [78] also supports this notion. Retrospectively, it is important to note the statement of Helen Pearson in a Nature editorial commenting on these data in 2005: “a jab against one strain might even aggravate an infection with SARS virus from civets or another species” [79].

After the first wave of D614G, several other SARS-CoV-2 variants have emerged until the rise of the Delta and Omicron variants. Indeed, key variations in the ADE epitope at positions 69 and 215 have probably protected patients infected with Alpha or beta strains from the ADE risk (Figure 3). Nevertheless, these variants also showed significant variability of the neutralizing epitope, which could have decreased vaccine efficiency [80]. The situation is more dramatic for the Delta variant. Indeed, several studies underscored the potential risk of ADE when a Delta SARS-CoV-2 variant infects a vaccinated individual [33,34]. Our study confirms this possibility and further extends it to other circulating variants, including lambda and mu, for which the neutralization/facilitation balance is unfavorable. A useful approach to anticipate such ADE risk in face of any variant is to analyze both the ADE and neutralizing epitopes of the NTD, as developed in Figure 3. At first glance, one can determine the balance between neutralization and facilitation and assess the risks of virus escape, ADE and/or both. Our molecular modeling approaches confirmed that hot mutational spots in ADE and neutralizing epitopes of the NTD give reliable information on antibody recognition of the spike protein, allowing us to determine which way the balance between neutralization and facilitation is tipping.

We recently hypothesized that the Delta variant has been dominating because its electrostatic surface potential of the NTD region that faces the host cell membrane has evolved to a large electropositive flat area that is complementary to the electronegative surface of lipid raft gangliosides [36]. The electrostatic potential surface value, which reflects the kinetics of virus infection, is a key parameter of a mathematic formula giving the transmissibility score (T-index) of any SARS-CoV-2 strain. This original and straightforward approach, which has recently received experimental confirmation for both enhanced transmissibility and faster infection kinetics [81,82], allowed us to correctly predict the rapid emergence of the Delta variant (T-index > 10) over Alpha (T-index < 4) even though both variants display a similar affinity for ACE-2 [36]. The T-index of the Delta variant is higher than the initial Omicron BA.1 strain (T-index < 5), but Omicron essentially uses the endocytosis pathway of infection so that it cannot be compared to other variants on the sole basis of the T-index [43]. Nevertheless, the electrostatic surface potential measured at the tip of the NTD (which faces lipid rafts), is a key parameter of the T-index that is submitted to evolutionary forces generating high mutation rates. This is not the case for the opposite side of the NTD whose genetic variability is limited. Overall, this retrospective analysis of 1,860,489 SARS-CoV-2 genomes reveals an early divergence between conserved (ADE) and variable (neutralizing) epitopes that appears to follow a biochemical logic controlled by virus-cell interactions.

## Figures and Tables

**Table 1:** Mutations in SARS-CoV-2 variants.

Virus Strain	NTD	Rod
Alpha α B.1.1.7 (UK)	∆H69 ∆V70 ∆Y144	D614G P681H T716I S982A D1118H
Beta β B.1.351 (S_Afr)	L18F D80A D215G ∆L242 ∆A243 ∆L244	D614G A701V
n/a C.1.2 (S_Afr)	P9L C136F ∆Y144 R190S D215G ∆A243 ∆L244	D614G H655Y N679K T716I T859N
Gamma γ P.1 (Brazil)	L18F T20N P26S D138Y R190S	D614G H655Y T1027I V1176F
Delta δ B.1.617.2 (India)	T19R T95I G142D ∆E156 ∆F157 R158G	D614G P681R D950N
Mu µ B.621 (Columbia)	T95I +143T Y144S Y145N	D614G P681H D950N
Lambda λ C.37 (Peru)	G75V T76I R246N ∆S247 ∆Y248 ∆L249 ∆250 ∆P251 ∆G252 ∆D253	D614G T859N
Omicron ο B.1.1.529 (S_Afr)	A67V ∆H69 ∆V70 T95I G142D ∆V143 ∆Y144 ∆Y145 L212I +214EPE	D614G H655Y N679K P681H N764K D796Y N856K N954K L981F

Mutation patterns in the NTD and rod-like regions of the SARS-CoV-2 spike protein were obtained from the GISAID database. Deletions (∆) and insertions (+) are indicated.

**Table 2:** Frequency of ADE and neutralizing epitope sequences.

Number of Mutations	1052 mAb (NTD) ADE 27-32 AYTNSF	1052 mAb (NTD) ADE 64-69 WFHAIH	1052 mAb (NTD) ADE 211-218 NLVRDLPQ	1052 mAb (Rod) ADE 600-607 PGTNTSNQ	1052 mAb (Rod) ADE 674---691 YQTQ---SQS	43-3-14 mAb (Rod) ADE 611-617 LYQDVNC	4A8 mAb (NTD) Neutralization 144-158 YYHKNNKSWMESEFR	4A8 mAb (NTD) Neutralization 242-249 LALHRSYL
0	98.99	94.35	99.13	99.92	99.40	<0.05	<0.05	99.22
1	0.98	5.46	0.82	0.05	0.06	98.70	3.47	0.36
2	-	0.15	<0.05	-	-	1.29	92.04	0.06
3	-	-	-	-	-	-	4.23	0.09
4	-	-	-	-	-	-	<0.05	0.22
5	-	-	-	-	-	-	<0.05	-

The frequency of mutations of each epitope sequence is calculated as the percentage of identity with the reference amino acid sequence of the SARS-CoV-2 spike protein (Wuhan strain). The most variable amino acid residues of each epitope are underlined. 1,860,489 sequences were analyzed from 1 June 2021 to 27 November 2021. The raw data were obtained from the Los Alamos database.

## Data Availability

The authors confirm that the data supporting the findings of this study are available within the article.
